# Bilateral ectopic third molars in maxillary sinus associated with dentigerous cyst—A rare case report

**DOI:** 10.1016/j.ijscr.2019.07.072

**Published:** 2019-08-02

**Authors:** Sarwpriya Sharma, Jaideep Singh Chauhan

**Affiliations:** a Consultant, Department of Maxillofacial Surgery & ‘Smile Train’ Cleft Centre CHL Hospitals, AB Road, LIG Square, Indore (M.P.) India; b Consultant & Head, Department of Maxillofacial Surgery & ‘Smile Train’ Cleft Centre CHL Hospitals, AB Road, LIG Square, Indore (M.P.) India

**Keywords:** Impacted, Maxillary molars, Ectopic, Maxillary sinus, Dentigerous cyst

## Abstract

**Introduction:**

Ectopic eruptions of a tooth in a non-dentate region is a rare entity and such cases have been reported to occur in the nasal cavity, orbital floor, maxillary sinus, mandibular condyle, chin, palate and coronoid process.

**Case presentation:**

We report a case of bilateral ectopic eruption of maxillary third molars associated dentigerous cyst. A 27 years old female patient reported to us with chief complaint of purulent discharge form nose and recurrent facial swelling for last 2 years. Complete removal of cystic lining and extraction of ectopic maxillary third molars along with mandibular third molars was carried out.

**Results:**

Post-operative healing was uneventful.

**Discussion:**

Bilateral ectopic eruptions of maxillary third molars in Antrum of Highmore (Maxillary sinus) are rarely seen and scantly documented in the literature. Dental ectopia may occur in antrum and present with local sino-nasal symptoms attributed to chronic sinusitis. Due to its rarity and lack of consensus over its management, the incidence deserves to be added to the literature & discussed.

## Highlights

• Bilateral ectopic eruptions of maxillary third molars in Antrum of Highmore (Maxillary sinus) are rarely seen and scantly documented in the literature.
• Due to its rarity and lack of consensus over its management, the incidence deserves to be added to the literature & discussed.
• An unusual case of bilateral ectopic third molars in maxillary sinus associated with dentigerous cyst, and impacted mandibular third molars.
• Ectopic teeth are commonly observed in the second or third decade of life. The age range varies from 4 to 57 with a mean age of 28.06 years.

## 1 Introduction

Ectopic eruption is a disturbance in which the tooth does not follow its usual course of eruption [1]. In most of the cases, tooth eruption process is a passive one, but an abnormal tissue interaction during odontogenesis may result in ectopic tooth development and eruption. Ectopic eruption may result due to one of the three processes: developmental disturbance, pathological process and iatrogenic activity [2]. Etiological factors may include developmental disorders such as cleft palate, trauma causing displacement of the teeth, maxillary infection, crowding, genetic factors, & high bone density. In this paper, we report an unusual case of bilateral ectopic third molars in maxillary sinus associated with dentigerous cyst, and impacted mandibular third molars.

## 2 Case report

A 27 Years old female patient reported to us with chief complaint of purulent discharge form nose and recurrent facial swelling for last 2 years. She experienced pain over right cheek region which was mild, intermittent and dull- aching in nature, she also noticed salty taste occasionally. She had a history of three-four courses of antibiotics prescribed by physician, but she had no permanent relief. Therefore, she was referred to our centre for management.

**Fig. 1:**
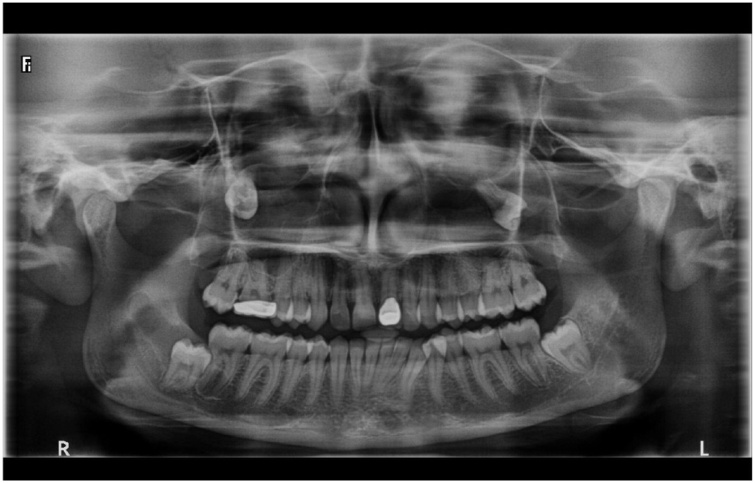
Orthopantomograph.

**Fig. 2:**
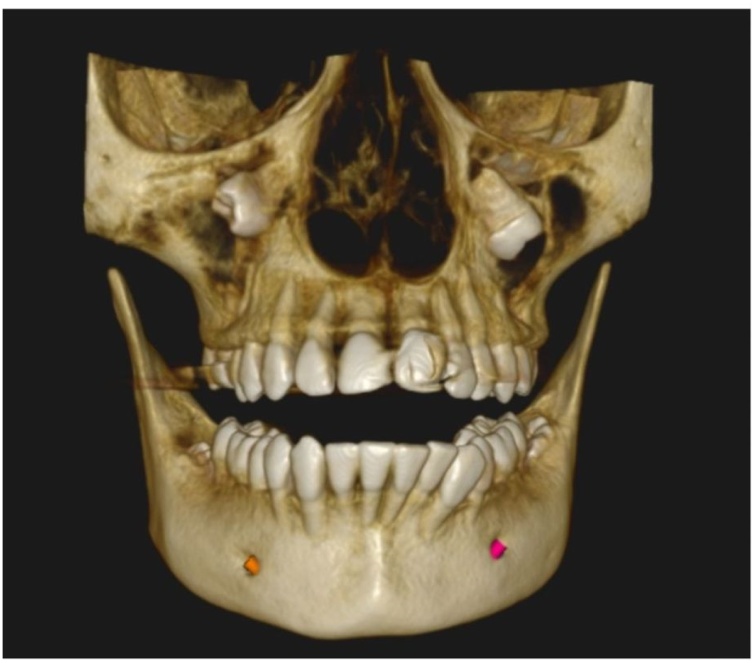
CBCT.

On clinical examination, mild facial swelling over right middle third of the face extending supero-inferiorly from infraorbital rim to the imaginary ala-tragus line and antero-posteriorly from right ala to body of the zygoma. Mild tenderness was present on palpation. Intraorally, other than missing maxillary third molars, no other significant findings were noted. Orthopantomograph (OPG) revealed ectopic maxillary third molars in the maxillary sinus along with impacted mandibular third molars (Fig. 1). In order to determine exact location of maxillary molars, since they were seen in close proximity to infraorbital rim, patient was advised Cone Beam Computed Tomography (CBCT). CBCT showed the presence of obliquely impacted 18 and 28, projecting into the maxillary sinus. (Fig. 2) Crown portions were located in maxillary sinus, whereas the root portions were in close approximation with medial wall of maxillary sinus. A hyperdense area was seen in the right maxillary sinus, suggestive of chronic right maxillary sinusitis and there was partial destruction of lateral wall of left maxillary sinus. CBCT scans confirmed the diagnosis of right maxillary sinusitis with ectopically erupted maxillary third molars.

On the basis of clinical and radiological examination, surgical removal of all impacted third molars along with cyst enucleation was planned through intraoral approach under general anaesthesia.

**Fig. 3:**
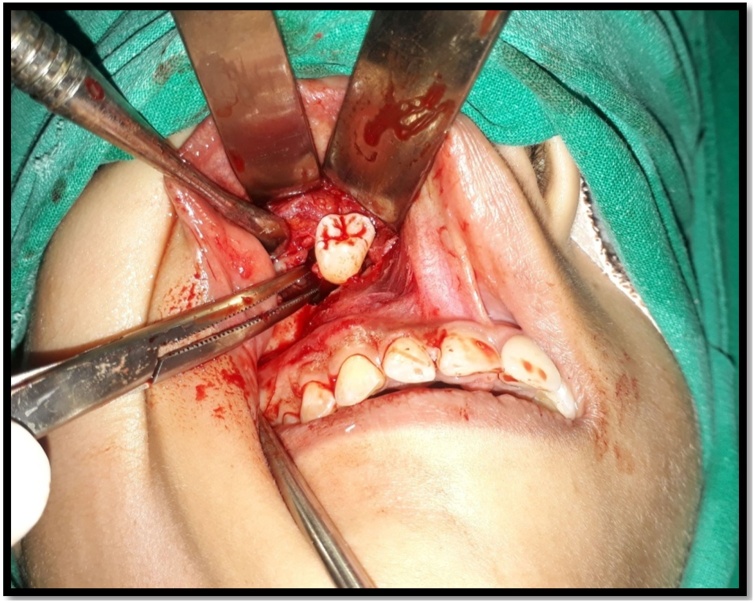
Intraoperative picture of 18.

**Fig. 4:**
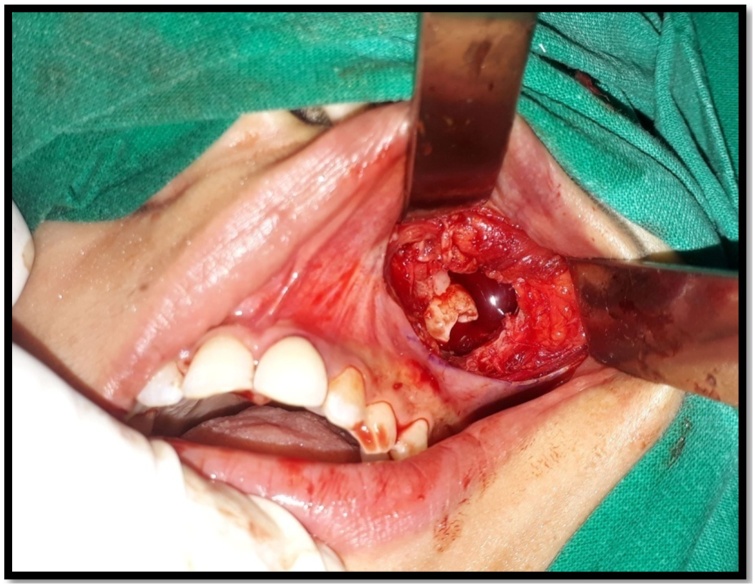
Intraoperative picture of 28.

**Fig. 5:**
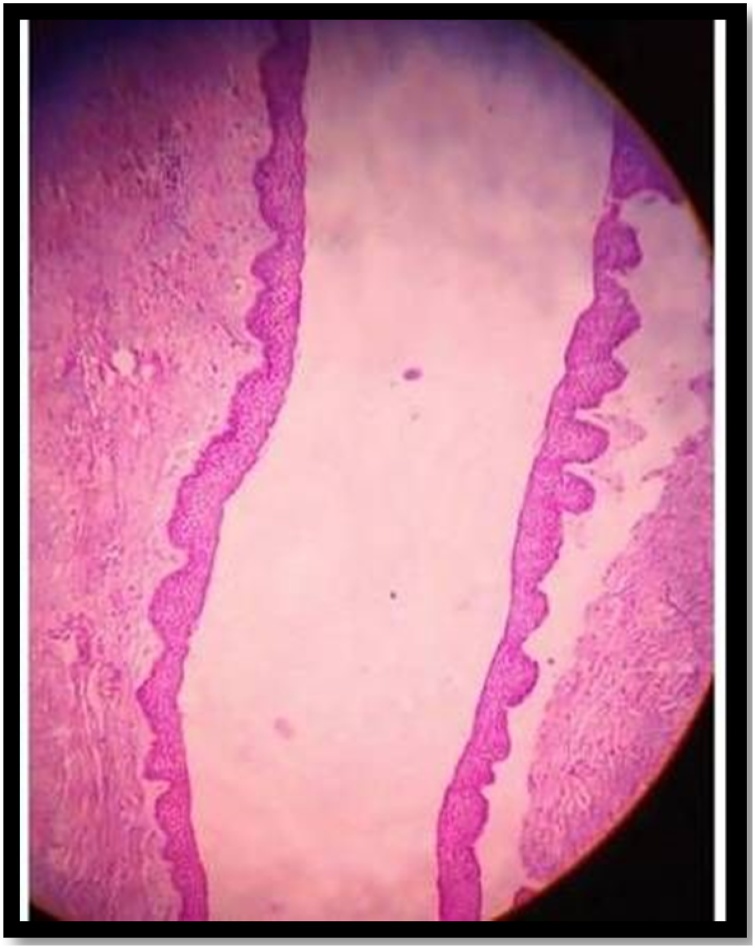
Histopathology.

**Fig. 6:**
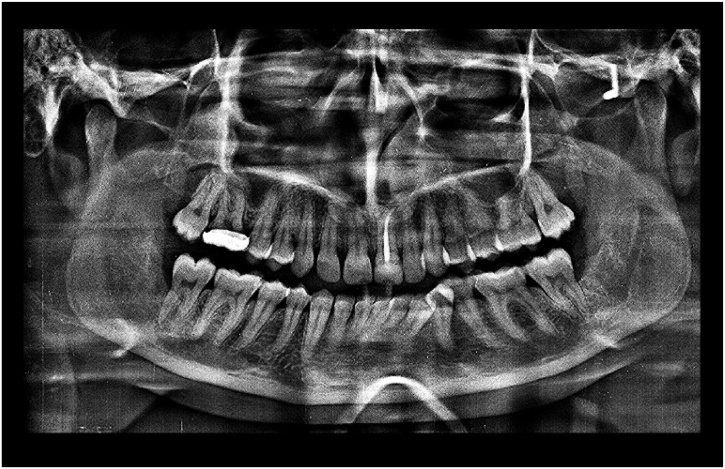
Six Months Postoperative Orthopantomograph.

Standard preparation was done. Under nasotracheal intubation, a vestibular incision was given from first premolar to second molar, a bony window was created in anterolateral wall of maxillary sinus. Cystic lining identified, pus was evacuated, swab was taken and sent for culture and sensitivity. Complete removal of cystic lining and extraction of ectopic maxillary third molars (Fig. 3 and Fig. 4) along with mandibular third molars was carried out. Antrum was properly irrigated. The specimen was sent for histopathologic examination. The histopathogical findings showed cyst wall lined by dentine epithelium with squamous and non-keratinizing epithelium which confirmed the diagnosis of dentigerous cyst with no signs of malignancy (Fig. 5) Post-operative healing was uneventful. (Fig. 6).

## 3 Discussion

According to Archer, Impacted tooth is, “The tooth, which fails to erupt in oral cavity in its functional position and which has lost its further potential of eruption. Maxillary third molars are the second most common tooth to be impacted, mandibular molars are the most common one. The third molar is the last tooth to erupt in the maxilla hence more likely to be affected by displacement while competing for space and this could be responsible for the high incidence of ectopic third molar in the maxilla. Odontogenesis begins in the sixth week in utero at the time of maxillary and mandibular dental lamina formation [3]. A series of complex tissue interactions between oral epithelium and underlying mesenchyme results in the formation of mature tooth comprising the crown and the root. Any abnormal tissue interaction during this may result in ectopic tooth development and eruption. Ectopic teeth are commonly observed in the second or third decade of life. The age range varies from 4 to 57 with a mean age of 28.06 years. The incidence is higher in men than in women [4].

Hamama et al. in the year 2018 [5], reported two cases of dentigerous cyst associated with an ectopic third molar in the maxillary sinus. Similarly, in our case, ectopic maxillary third molars were associated with dentigerous cyst bilaterally. The term “dentigerous cyst” was coined by Paget in 1853. These cysts are the most common type of developmental odontogenic cysts arising from the crowns of impacted, embedded, or unerupted teeth. The most reasonable theory to explain its pathogenesis appears to be that the cyst is the result of the accumulation of fluid between an unerupted tooth and the surrounding reduced enamel epithelium. About 30% of the dentigerous cysts occur in the maxilla. If a dentigerous cyst associated with ectopic tooth located in the maxillary sinus, symptoms usually occurs little late. It can cause local sino‑nasal symptoms like nasal obstruction, purulent rhinorrhoea, facial fullness, headache, hyposmia, and recurrent chronic sinusitis, elevation of the orbital floor. Extension of the lesion into the orbital floor can cause diplopia and possibly even blindness [6]. In our case, patient presented with chief complaint of purulent nasal discharge, recurrent facial swelling, and headache occasionally.

Radiological examination is essential for diagnosing the presence of an ectopic tooth. Advanced imaging modalities are very useful for the determination of exact location of tooth and its surrounding anatomical structures. Cone beam computerized tomography (CBCT) and CT scans are certainly superior to panoramic radiographs. Both gives better precision in localization of pathology. CBCT is also useful to delineate the three dimensional morphology of the ectopic tooth, its inclination and proximity to the sinus which aids in surgical planning.

The treatment of choice of ectopic teeth associated with cystic lesion in maxillary sinus is surgical removal of the tooth along with enucleation of the cyst. Various techniques have been discussed in the literature including intraoral approach, extraoral approach, and endoscopic procedures. Di Pasquale and Shermetaro used a nasal endoscope to create a middle meatal antrostomy and deliver the ectopic tooth and its cystic contents [7]. Hamama et al., [5] used midfacial degloving approach to for extraction and enucleation of cyst. In our case, we used an intraoral approach for removal of tooth and enucleation of cyst, as the patient was young and concerned with cosmesis.

Written informed consent was obtained from the patient for publication of this case report and accompanying images.

## Sources of funding

None.

## Ethical approval

Yes, Ethical approval has been taken.

## Consent

Yes, written informed consent has been taken for procedure, publication as well as for accompanying images.

## Author contribution

Dr. Sarwpriya Sharma & Dr. Jaideep Chauhan, both the authors contributed equally to the paper which includes Surgery, design of case report, writing, interpretation etc.

## Guarantor

Dr. Vilas Newaskar, Consultant and Head, Department of Oral & Maxillofacial Surgery, GDC Indore (MP).

## Provenance and peer review

Not commissioned, externally peer-reviewed.

## Declaration of Competing Interest

No Conflict of interest.
